# Mr. Bullin’s Tribute to America

**Published:** 1888-02

**Authors:** 


### "MR. BULLIN'S TRIBUTE TO AMERICA."

All the American Journals are cock-a-hoop over the "tribute" paid in the now well-talked of Chester address; one would imagine such jubilation followed upon restitution of some long contested and oft refused right, instead of a mere passing notice of our indebtedness to our cousins, a debt which has been cheerfully conceded, but not quite in the expansive manner sometimes indulged in by after-dinner speakers.—*British Journal of Dental Science.*

Will the B. J. D. S. kindly indicate *what* American Journals are "cock-a-hoop" over Mr. Bullin's address, or, indeed, what journals have particularly referred to it in any manner ?

The address at Chester and the discussion to which it gave rise are matters which concern English dentists alone, and American journals are not apt impertinently to interfere in family quarrels. Especially do we believe American dental journals quite incapable of an attempt to make party or controversial capital out of an unfortunate difference of opinion among our English brethren, or to misrepresent facts in order to score an unfair point against an adversary.

